# Gendered Justice in China: Victim–Offender Mediation as the “Different Voice” of Female Judges

**DOI:** 10.1177/0306624X20936202

**Published:** 2020-07-02

**Authors:** Shuai Wei

**Affiliations:** 1 University of Cambridge, UK

**Keywords:** victim–offender mediation, judges, gender, propaganda

## Abstract

Attempts to uncover the “different voice” of female judges through testing the statistical significance of judges’ gender in decision making have offered inconsistent results. Meanwhile, a proliferation of research suggests that such “voice” might be detected through qualitative analysis. Existing findings indicate that when female judges have discretionary power regarding case management, they will typically foster a process of settlement. Based on this information, I conducted eight months of fieldwork in China and observed 68 victim–offender mediations in four district courts. I found that the criminal division is widely perceived as a masculine setting, and female judges are accustomed to employing mediation as a preferred dispute resolution method to facilitate reconciliation between the two parties and seek civil compensation for victims. Such judicial behavior is a result of propaganda from the Supreme People’s Court and a reflection of female judges’ life and work experience. By contrast, a neglect of mediation among male judges can be identified in the same workplace. The belief that mediation is feminine and time-consuming contributes to this neglect. In addition, rape lawsuits are an exception for mediation. This explorative research not only represents one of the first efforts to reveal a “different voice” in the Chinese criminal justice system but points out a direction of research for studying the judicial behaviors of female judges worldwide.

In Shakespeare’s *The Merchant of Venice*, Portia, disguised as a man (which is the only way she can argue the law), assumes the role of a lawyer’s apprentice and eloquently pleads for the life of Antonio before a judge. Portia is perhaps one of the first female “lawyers” to be named in Western culture, but her story indicates that the early legal society was built as a mostly male field, macho, and hierarchical. It is true that the legal profession historically has been homogeneous in gender^1^ and ethnic make-up, greatly impacting the resulting power structure (Brockman, 2001; Kronman, 1995). In such institutional settings, many sentencing statutes and judges in the United States make gender distinctions that are transmitted into disparate sentences for male and female offenders found guilty of the same offense (Armstrong, 1977). Nearly three decades ago, the 1995 Beijing Declaration and Platform for Action (article 142, page 58) specified that government should “ensure that women have the same right as men to be judges, advocates, or other officers of courts, as well as police officers and prison and detention officers, among other things.” Even in the 21st century, it is still easy to imagine a time when bringing a dispute before a court would certainly mean a white male judge sitting on the bench and determining the outcome of the litigation. Over the years, feminist legal scholars have continuously challenged the “rationality” of laws and legal systems (Fineman & Thomadsen, 2013). They understand maleness as a social and political concept, not a biological attribute (Littleton, 1986). It is men who define objectivity and neutrality, pushing women to see reality in those terms. MacKinnon (1983) thus argued that “justice will require change, not reflection” (p. 658).

Feminist legal scholars have also campaigned for gender equality and diversity in a judiciary which has long been perceived as “pale and male” (Rackley, 2012, p. 7). Research has revealed that women may be able to engage in agenda-setting for women’s equality and rights when they constitute a critical mass in political bodies (Bratton, 2005). As a result, a rich body of international literature arguing for a strong role for women in the administration of justice has been produced (Schultz & Shaw, 2013). The scope of these research outputs not only covers the methods of selecting judges in many parts of the world, but also touches on the advancement of judicial careers for female judges. One line of this inquiry examines the role of gender in the application of the law, and scholars have wondered whether women can bring something different to the adjudication process based on their life and work experience. The suggestion that female judges rule “differently” from their male colleagues is typically tied to the work of Gilligan (1982). In her book *In a Different Voice*, Gilligan identified two distinct “voices,” or reasoning processes, corresponding (in her research subjects) to men and women. She argued that the woman’s voice is not deficient (as was assumed by dominant psychological theories of the time), but is merely different from the mainstream (masculine) voice. Soon after the publication of Gilligan’s book, her work was heavily criticized on methodological grounds because of her extremely small, selective sample (Auerbach et al., 1985). Given the complexity of social reality, other critics also argued that one should be careful not to attribute too much influence to gender alone in decision making, but rather explore a combination of variables such as professional training and the prevailing political regime along with personal attributes, such as age and marital status (Sonneveld & Lindbekk, 2017). While Gilligan had likely not intended to provide a conceptual framework for advocating the inclusion of female judges in the judiciary, this is precisely what happened following others’ interpretation of her work (Kenney, 2008; Minow, 1987).

The research literature reveals that in divorce disputes, sex discrimination and sexual harassment lawsuits, same-sex rights cases, and asylum-seeking applications, many female judges in the United States have different jurisprudential philosophies from their male colleagues (Boyd et al., 2010; Coontz, 2000; Davis et al., 1993; Martin & Pyle, 1999, 2004; McCall, 2003; Ramji-Nogales et al., 2007; Smith, 2004; Songer & Crews-Meyer, 2000; Songer et al., 1994; Spohn, 1991). However, some scholars have also revealed a more homogenous picture (Gottschall, 1983; Gruhl et al., 1981; Johnson, 2006; Kritzer & Uhlman, 1977; Walker & Barrow, 1985). Accordingly, there is very little consistent statistical evidence for an essential difference between male and female judges in decision making. Qualitative studies on the gender effect in judging are scarce but have uncovered a “different voice” (Miller & Maier, 2008). For instance, Artis (2004) conducted in-depth, face-to-face interviews with 25 trial court judges in Indiana and investigated their accounts of whether they continued to use the tender years doctrine in custody disputes, even though the custody statute is explicitly gender neutral. She found that female judges are less likely to support the tender years doctrine than male judges.

Since women began entering the legal profession in significant numbers, mediation has been gaining recognition as a viable process for resolving legal disputes. As Galanter (1985) puts it, settlement is now “a respectable, even esteemed, feature of judicial work” (p. 257). Applying Gilligan’s (1982) ethic of justice and ethic of care to the dispute resolution process, proponents of a care-based approach regard mediation as a process that fulfills many of the characteristics of the ethic of care (Klein, 2005). First, a court battle tends to breed hostility and reinforce anger between disputants because they are increasingly polarized in the adversarial process. In contrast, mediation defuses anxiety by promoting cooperation between the parties in a structured and supportive process. Second, mediation could help the parties to identify important issues between themselves and produce an agreement for the future that both sides can accept. Third, in the criminal justice system, mediation offers the opportunity for offenders to apologize to their victims, expressing remorse. Oftentimes, victims’ need for a simple apology from offenders is greater than their need for retribution through litigation (Goolsby, 1990). Because of these merits, mediation “promised to be a feminist alternative to the patriarchally inspired adversary system,” which is arguably the most trenchant critic of alternative dispute resolution (Nader, 1993, p. 10).

Exploring a “different voice” in mediation, Boyd (2013) identified that female judges successfully fostered settlement in their cases more often and more quickly than their male colleagues, using a data set of 18,000 civil rights and tort cases in four federal district courts across 9 years. She pointed out that the prerequisite for such differences in settlement is the discretion that lies with individual district judges to determine how hands-on they wish to be in supervising their cases and how informal in mediating disputes assigned to them. It is here that gender is perhaps most likely to play a role in determining whether judges take the initiative to actively promote settlement. It should be highlighted that female judges’ preference for mediation is not unique to Americans. Based on interviews and surveys with 31 Swiss judges and 80 other members of the legal profession, Ludewig and LaLlave (2013) found that female judges in Switzerland ranked mediation significantly higher than male judges did, and this difference was statistically significant. These findings seem to suggest that women do not leave gender at the door on entering the legal profession. Rather, it is an essential factor in their practice of law. It is one of the reasons why female judges are “more willing to try facilitation and are often better at it” (Klein, 2005, p. 785).

Gilligan’s theory and the gender effect on mediation have also helped to shape the debate on how Chinese women’s involvement in the legal profession might impact on the justice system. Scholars have found that the Chinese national role models for female judges, such as Chen Yanping, Jin Guilan, and Shang Xiuyun, are experts in mediation without exception (Song & Liu, 2016; Song & Xie, 2014). The fieldwork in two district courts of China carried out by Wei and Xin (2013) also illustrates how female judges understand the nature of their work in divorce mediations by following mediator settlement strategies, similar to those observed by Silbey and Merry (1986), to defuse domestic conflicts. Nevertheless, victim–offender mediation,^2^ an essential component of criminal trials, is an area that has never been touched on by researchers. According to article 101 of the 2018 Criminal Procedural Law, a victim has the right to file an incidental civil claim during the course of a criminal proceeding if he or she has suffered material losses as a result of the offender’s criminal act. A key assumption for such “voice” to emerge in victim–offender mediation is that, depending on the complexity of the case and their schedules, judges in criminal divisions have the authority to decide whether a case should go directly to trial or go through mediation for civil claims first. There is also no limit on the number of times such mediation can be attempted. However, according to article 122 of the 2017 Civil Procedure Law, judges do not possess such discretionary power in all civil claims because mediation should be used first unless it is refused by the parties. Echoing the findings on female judges’ discretionary power regarding case management and preference for settlement, this study aims to uncover such “voice” in the Chinese criminal justice system and thus to fill this research gap (Boyd, 2013; Ludewig & LaLlave, 2013). This article proceeds as follows: Part I offers an overview of the Chinese judiciary and argues for its gendered construction since its establishment. Part II explains the philosophical influence of Confucianism on Chinese people’s preference for mediation and reviews the literature on victim–offender mediation in China. Part III details the research design of my eight months’ fieldwork in four district courts of China, and Part IV presents major findings. Part V discusses theoretical and practical implications of these findings, and the last part identifies the limitations of the research and suggests two directions for further study.

## Chinese Judiciary and Its Gendered Construction

The Chinese state has historically played an overwhelmingly important role in defining the criteria for performing gendered identities (Mann, 2011). Prior to the late 1970s, the government disseminated a Marxist ideology that promoted egalitarian gender roles (Davis & Harrell, 1993). In this communist version of modernity, women’s liberation is achieved primarily through mass labor participation and, to a lesser degree, through mass education (Ji, 2015). Unfortunately, the revolution stopped short of the private sphere of the family, and, as in many Western contexts, traditional gender norms and a gendered division of labor in the home remain largely unchanged in China (Parish & Farrer, 2000). Alongside the rapid economic reformation and modernization starting in the late 1970s, China has witnessed a resurgence of patriarchal Confucian traditions (Dutton, 1992). This is because the post-1978 economic reform prioritized economic development and capital accumulation over the development of women as productive members of society (Leung, 2003). As a result, it occurred in a way that dismantled some of the policy arrangements that ensured the equality for women in the Mao era. The foundation of women’s political participation was thus seriously damaged (Min, 2011).

Female judges were initially a minority in the judiciary for historical reasons, including the fact that in the beginning judges were chosen from retired military personnel, who were largely male (Shen, 2017). Therefore, the reason for the previous rarity of women in the Chinese judiciary differs from that of women’s underrepresentation in courts of the Western world. However, like many other countries, China is experiencing a rapid process of feminization in legal education and legal profession in the early 21st century. In 2010, there were approximately 45,000 female judges in China, accounting for about a quarter of all judges in the country. By 2013, the number had increased to about 57,200, or 28.8% of all Chinese judges (National Bureau of Statistics of People’s Republic of China, 2015). In 2017, female judges constituted 32.7% of all judges nationwide, which was 21.7% higher than in 1982 (State Council Information Office, 2019). In the United States, women are often concentrated in the public sector of the legal profession, whereas men occupy most of the lucrative partner positions in corporate law firms (Dinovitzer & Hagan, 2014). The situation in China is not so different: Women are more likely to be found in courts and procuratorates than in law firms, and they are less likely than men to move from the public sector to the private sector of the profession (Zheng et al., 2017). Nevertheless, female judges suffer from a gendered career mobility issue in the Chinese judiciary, and Zheng et al. (2017) named such issue as the “elastic ceiling.” In their study of 55 judges from four provinces and one municipality of China in 2012 and 2013, Zheng et al. (2017) found that although female judges have made notable progress in weakening the glass ceiling in the judicial hierarchy, their upward mobility often stops at the mid-level leadership, such as division chiefs and vice-chiefs. High-level leadership positions such as court presidents and vice-presidents are still dominated by men.

In recent years, female law school graduates effected a large-scale entry into the Chinese legal profession, and a significant number of those with graduate degrees in law passed the national judicial examination and civil service examination for vacancies in courts (Zuo, 2018). Female graduates who chose to work in criminal divisions might be assigned to work in the juvenile section, a subdivision of the criminal division, and they have frequently been portrayed by the media as “mother judges” (*faguan mama*) because they are women dealing with offenders aged 14 to 18 (Zhu, 2012). Normally, trials in the juvenile section are less formal, and allow more participation by the children and their family. With rare exceptions, the role model female judges in the juvenile section are reported to devote themselves passionately to preventing youth offending and reoffending (Song & Liu, 2016). Even though some female judges choose to stay in the juvenile section after a few years, most choose to change post to hear normal criminal cases.

In addition, the *People’s Court Daily*, a propaganda mouthpiece of the Supreme People’s Court (hereinafter SPC), often describes female judges who work in criminal divisions as “different” in many ways from their male colleagues. For example, Yu Huafen, a criminal division chief in the Xuanwei district court and a role model for female judges, is said to “perfectly handle her femininity in a masculine court environment” (Tong & Tang, 2015, p. 5). The *People’s Court Daily* has asserted that Yu’s achievements, as the judge who settled the highest number of criminal lawsuits in her division over 27 years, can be attributed to her combination of “feminine” characteristics, such as attention to detail, and her “masculine” workaholic lifestyle. The *People’s Court Daily* has also reported on the extent to which female judges’ working style in criminal divisions is “different” from male judges’ approaches. Zhou Xin, a model female judge from the criminal division of the Shanghai Intermediate People’s Court, was accustomed to speaking with victims’ relatives after trials and taking their opinions into consideration before reaching a decision (Yang, 2016, p. 5). Judge Zhou believed that, in addition to the adversarial court hearing, this was the only way to reveal and understand the full story. Although stories reported by the *People’s Court Daily* may not accurately and critically reflect female Chinese judges’ actual working style in criminal divisions, they could be referenced as a starting point for comparative research on judges’ gender and judicial behaviors in the Chinese criminal justice system.

## Victim–Offender Mediation in China

The philosophical influence of Confucianism has been pervasive among the Chinese people from ancient to contemporary times. It is perhaps unsurprising that the pervasive teaching of Confucius has left the traditional Chinese culture and discourse with a non-litigious outlook (Cohen, 1966). As Wu (2014) pointed out, “probably nothing can disturb harmonious relationships more than lawsuits in an acquaintance society” (p. 117). Hence, the traditional Chinese method of dealing with disputes inevitably lies in mediation. In fact, one may go so far as to observe that it is “dispute dissolution rather than dispute resolution which [the Chinese] hold dear” (Goh, 2016, p. 9). Another relevant social value is the notion of yielding (*rang*). The notion of yielding is positive: A party in a dispute forsaking something might, in another way, acquire something else. Yielding also suggests that one has the ability to look within oneself and acknowledge that one may not be completely faultless. The Chinese cultural desire for yielding is the prime motivating factor behind the need for compromises, which, in turn, shapes the process of any mediation (Bodde & Morris, 1967). It is worth noting that women are perhaps the ones frequently on the side of yielding. This is because women were significantly more likely to tolerate disputes rather than to actively solve them by informal mediation or legal channels (Zhuo & Cao, 2016).

In the four decades since China’s implementation of the reform and opening-up policy in 1978, the country’s economy has developed rapidly, and income inequality has increased significantly (Yao, 1999). With rising income inequality has come political corruption and official misconduct, including within the justice system (Lewis & Litai, 2003; Xiong & Wei, 2017). As a result, increasing tension and strong resentment of “the rich” and “the privileged” has translated into waves of social unrest and street protests against local governments, which represent the Communist Party of China (hereinafter CPC; Su & He, 2010). Social survey data indicate that among people who have experienced grievances or disputes, their preferred way of addressing these is by the administrative remedy, either through local government or semi-government agencies (Jiang & Wu, 2015). To ease the conflict between citizens and the state, in October 2006 the CPC introduced the political objective of building a “harmonious society.” Scholars have understood this as a process through which social stability might be achieved (Li, 2017). This political agenda has also “reframed the approaches to law and justice to fit the contours of the stability imperative” (Trevaskes et al., 2014, p. 2). In the judiciary, the SPC issued a judicial opinion in 2010 encouraging lower courts and judges to employ the Ma Xiwu adjudication method, which focuses on mediation, as a preferred approach for resolving disputes. In practice, this opinion was translated into concrete moves for judges to avoid issuing decisions that might result in mass protest and petitions to higher authorities (Minzner, 2011). The adoption of mediation has commonly been viewed by scholars as a move toward “restoring the age-old Confucian ethics of societal balance and harmony” in the legal system (Weatherley & Pittam, 2015, p. 279).

Although mediation has been typically used in non-criminal proceedings, it is also appropriate for criminal cases in which a civil claim for compensation is brought to court by a victim. Victim–offender mediation was originally designed for cases where offenders have committed minor criminal acts but demonstrated remorse during the investigation and prosecution processes. It seems to have been first used by a Beijing district court in 2002 in cases of assault with minor injury (Xiang, 2013). After a few years of testing this method, such mediation became a widely accepted practice within the police, the procuratorate, and the courts. In 2012, it was formally stipulated in the Criminal Procedure Law that criminal acts such as common assault, theft, deception, and illegal trafficking can be handled through victim–offender mediation. In 2016, the scope has been expanded by the SPC to cases involving assault (including assault with serious injury), serious traffic offenses, intentional destruction of property, robbery, theft, fraud, rape, extortion, and negligent homicide, with malfeasance as an exception. Some serious crimes, for which offenders face a sentence of imprisonment of three to seven years, can also be handled through victim–offender mediation. Assault, theft, and reckless driving are normally the top three crimes subject to victim–offender mediation across the country (Lu et al., 2017; Yuan, 2017).

One merit of introducing victim–offender mediation to the criminal justice system is that mediated cases are less likely than adjudicated ones to result in petitioning to a higher level of the administrative and judicial systems (Liebman, 2011). This is because the purpose of such mediation is to heal the harm done by offenders, work out a plan to compensate victims, and restore the community in full. In mediation, offenders’ financial compensation of victims is considered a sign of sincere apology and repentance, an act of good will to mend the broken relationship, and a form of restitution (Lu et al., 2018). According to article 9 of the 2017 Sentencing Guidelines stipulated by the SPC, offenders will receive a 30% deduction of their sentence if they actively compensate the victim but fail to obtain the latter’s forgiveness; a 40% deduction will be applied if the offender has actively compensated the victim and received forgiveness. Judges are able to grant a 20% deduction if the victim has forgiven the offender without compensation. Evidently, the offender’s attitude and compensation in mediation has frequently impacted judicial decisions.

There have been some criticisms of victim–offender mediation as it is short on formal procedure and lacks oversight (Jiang, 2017). It has also been criticized for undermining the integrity of criminal justice system because in practice the offender is allowed to avoid criminal responsibility through financial settlement, although this could be an unintended consequence (Yuan, 2018). Take Henan province as an example: High compensation is not only common, in some cases it is ten times the statutory standard (Xiang, 2013). Hence, offenders without financial resources must choose between paying to reduce prison time and saving money but serving the sentence. Mediation may also considerably increase judges’ workload. Song et al. (2009) revealed that the average time spent on each mediation was around two hours, excluding the time spent on preparing the mediation and writing the final agreement. Because of the workload pressure, judges must be efficient in reaching a settlement for both parties. In her five-month period of fieldwork in an eastern Chinese city, Yuan (2017) even found that some judges had no time to listen to the basic facts from the opposing sides in mediation.

## Research Design

Contemporary China provides a strategic setting for criminological research because the country has experienced profound social change and rising levels of crime since it implemented the reform and opening-up policy in the late 1970s. The purpose of the current inquiry is to gain knowledge of the perspectives and attitudes of judges involved in victim–offender mediations in their natural settings, and it demonstrates the feasibility of conducting such research in China despite the significant political, social, and cultural barriers. In order to complete this project, from June 2017 to February 2018, I spent 2 months each in District Court A in Shanghai, District Court B in Shenzhen, District Court C in Baoding, and District Court D in Shenyang. These four cities are located in different regions of China and differ in various aspects, such as population and leading industries. For example, Shanghai is a coastal metropolitan city in eastern China with a population of 24.18 million. It is also a global financial center and transport hub with the world’s busiest container port. Baoding, however, is a medium-sized inland city in northern China with a population of 10 million. Baoding has one of China’s biggest plants manufacturing the blades used in wind turbine generators, and also has good connections to other cities, being located on one of the main routes in and out of Beijing. Over eight months of fieldwork in four district courts, 68 mediation cases were observed, mostly involving traffic accidents and reckless driving (23 in total), assault (18), theft (13), robbery (9), and fraud (5). Since I guaranteed my interviewees anonymity and confidentiality, I took rigorous measures to protect all information that could identify the judges and specific places where interviews were undertaken. A table providing the aggregated information of the 42 participants of this study has been provided as an Appendix of the article. It is expected that this research represents the perspectives of these judges.

The difficulties of conducting empirical research into criminal matters in China have long been acknowledged and gaining access to respondents is particularly challenging (Curran, 2010). Although the conditions are improving, the obstacles encountered by researchers have changed little in 40 years (Heimer & Thøgersen, 2006). The first difficulty of conducting criminological research in China is political sensitivity. Traditionally, criminological research and data have been considered highly sensitive because the CPC believes that crime has no place in a socialist society (Johnson, 1986; Troyer, 1989). In addition, the CPC fears that publicizing and revealing crime-related information, which is guarded as a state secret, would tarnish the image of the nation and be “a source of international embarrassment” (Bennett, 2004, p. 12). Undoubtedly, familiarity with the political and social structures of Chinese society is essential to overcoming this obstacle. Yuan (2017) noted that the use of a less sensitive topic that could be tied to current state-promoted practices increases the likelihood of the researcher being accepted. I found that judges were pleasant when I shared my research objective on gender, law, and judging with them. The saying “women can hold up half the sky” (*funv nengding banbiantian*), a proclamation made by Chairman Mao in 1955 and a popular element of the CPC’s propaganda ever since, was frequently uttered by my hosts in first meetings. In District Court A, the deputy president even claimed that the era of “whatever male comrades can accomplish, women comrades can too” in his court is already a thing of the past. He observed that female judges in the criminal division consistently outperformed male judges, and he was proud of this. As a result, neither of my local hosts ever challenged me on the sensitivity of my research project.

The second hurdle to the collection of original data in China is access to criminal justice institutions. Researchers in Western countries frequently gain access through personal connections. *Guanxi*, the Chinese personal connection, is a form of social capital consisting of an individual’s instrumental and affective bonds in a complex social network governed by trust and reputation (Wang, 2014; Zhang et al., 2009). Given that Chinese people pay more attention to *guanxi* in exchanging favors and sharing resources reciprocally, gaining access to the field will depend, among other things, on whether the researcher has a strong, special relationship with the institutions to be researched. District Court A was my first destination, and I was introduced to the deputy president by a law school professor in Shanghai: The deputy president was a second-year PhD candidate of the professor. Therefore, my entry into the field depended entirely upon my connection to this professor and her connection to this deputy president. However, I was fully aware that good relationships established with one key respondent do not always extend beyond the individual contact and into the institution itself. Efforts must be made to build the relationships in order to gain the trust and confidence needed to be granted interviews and observations. During my two-month stay in Court A, I provided assistance to the criminal division chief while collecting data for my own research. The workload exceeded my expectations and took up much of my time in the first weeks. The chief understood this and did me favors, such as facilitating contact with other judges for interviews or asking his assistant to find me materials held by the division. My connection to District Court C was built on a long-term friendship with the division chief. This strong tie helped me to gain access to the criminal division, and I had opportunities to build mutual trust with his colleagues. As a gesture of good will, I provided help and assistance in writing notes and reports for them. My assistance in their work reinforced our personal relationships and facilitated the data collection process. Access to District Courts B and D was obtained from friends who work in the local judges’ colleges.

The third obstacle to conducting criminological research in China is the resistance from judges, an elite group whose counterparts in the West are known to be hard to reach. In the UK, for instance, “members of the senior judiciary in particular have never been in the least enthusiastic about research, frequently viewing such endeavors as an unwarranted intrusion into matters that should be their business and no one else’s” (Baldwin, 2000, p. 237). This is also the case of Chinese judges, although they do not enjoy the same status as their counterparts in the West. Shen’s (2017) fieldwork conducted in 13 courts in one province found that it is not only hard to obtain gatekeepers’ approval, it is challenging to persuade individual judges to take part in research activities when access has been authorized at the top. Shen correctly pointed out that, fundamentally, Chinese judges fear losing their jobs as a consequence of participating in academic activities because of the lack of judicial independence. Since judges are concerned that their career could be jeopardized by the outcomes of research projects, they are expected to turn down requests for interview—especially requests from scholars with institutional affiliations to foreign universities. In this project, I found that having Chinese nationality and student status significantly reduced judges’ resistance. In Court A, the deputy president introduced me to judges in the criminal division as “a student who is eager to learn.” In Court C, the division chief told his colleagues in his introduction that “students only know the law from books, so we should teach him what the law in action is.” Both the deputy president and the division chief were correct about my motivation on the field trip: I was driven to observe victim–offender mediations and speak with judges about their thoughts on such behaviors. Their introduction of me as a student, instead of as a researcher from a university overseas, alleviated judges’ concerns about my “intrusion” into their workplace. I also understand that their positive responses and support for my fieldwork were the outcome of *guanxi*.

The basic assumption of this research is that judicial attitudes are related to judicial behavior, and such attitudes are the key factor in explaining judges’ motivations in using victim–offender mediation. Therefore, I used interview and observation as major research methods. All interviews were undertaken following a semi-structured schedule; they were not recorded since recording devices are explicitly prohibited on court premises. As a result, all data are based on brief notes taken during interviews and filtered through my memory. The transcriptions were authenticated by judges, and in some cases a follow-up interview clarified or expanded themes which had arisen in the first interview. In addition, I chose to incorporate information gathered from direct observation of victim–offender mediation as evidence for analysis, because direct observation is “one of the primary data collection methods for naturalistic or fieldwork settings” (Gray, 2013, p. 185). Nevertheless, observation of this kind runs the well-documented risk that the presence of the observer will affect the behavior of those who are being observed (Bottoms, 2000). To overcome this problem, I spent four weeks in an immersion period in each division before starting to observe. During this period, I found opportunities during lunchtimes to speak with judges on common topics and shared experiences. I also managed to explain my research project in detail during the division’s business trips. These interactions positively sped up the immersion process and meant that I was more likely to obtain research data rather than presentational data through interviewing and observing judges. Judges who were being observed were comfortable with my presence and behaved naturally after the assimilation.

## Major Findings

During my time in the four district courts, I sensed that the criminal division is a masculine setting for female judges, and pretrial mediation is frequently used by them to settle conflicts between victim and offender, compensate the victim properly, and reach mitigating conditions for offender. Regarding the masculine atmosphere, my interviews suggested that when recruiting judges for certain positions, a male preference has been stronger in certain divisions. The top three on the list are enforcement, security, and criminal division. Enforcing judicial decisions requires frequent travel and is sometimes confrontational, so it is generally considered a job for men. Court security officers often need to protect criminal suspects in trials and secure the safety of any person in the building, so this is also deemed a job for men. The reasons why women are not suitable for jobs in criminal divisions shared by interviewees include frequent encounters with “horrifying” criminal suspects and “bloody photos” of murder cases. Court officials also expressed concern for female judges’ personal safety—judges in criminal divisions sometimes face threats or even physical attacks from resentful litigants, especially after making “unfavorable” sentencing decisions. A deputy division chief (A-F-1)^3^ who has worked in District Court A for over two decades shared some reflections on her early experience in the criminal division:

On my first day in this division in 1997, I felt like I was married to eight husbands—they never treated me as their colleague, but as their secretary. They relied heavily on me to handle the administrative business in our division, so I barely had time to hear trials. Also, a “paternalistic” atmosphere pervaded in this division, as if they had to help me a little bit more in everything because I am a woman.

I also observed how female judges in criminal divisions mediate civil claims. When they have an opportunity to handle victim–offender mediation, female judges often propose a compensation sum that is twice (in extreme cases, three times) the statutory standard for offenders to pay, because they expect compensation beyond victims’ expectations to encourage them to quickly agree to a deal. Female judges also request that offenders pay the medical expenses of victims first, frequently observed in traffic accident mediations in which the victims’ family members are particularly concerned about medical costs. Once medical expenses have been paid, judges expect offenders to cover additional costs, such as the living expenses. These two expenses were found to attract particular attention from judges since they make decisions on offenders’ sincerity based on their approach to such payments. In cases in which offenders are sincerely remorseful but too poor to compensate victims, judges ask the victims’ family to accept monthly letters from offenders about their progress in prison. If this strategy is ineffective, judges ask the offenders’ relatives to provide help to the victims’ family. This approach may not always work in mediation, but judges observed used it frequently when offenders’ financial capacity was limited, and judges still attempted to reach a consensus between the two parties. Oftentimes, the notion of yielding plays an important role in the process of settlement. Not surprisingly, the restoration of relationships in victim–offender mediation is translated into apology, forgiveness, and compensation in practice.

It is also essential to note that a special feature of Chinese judicial mediation is that such mediation is part of judges’ formal role, thereby giving them greater authority and more power to intervene. Because of judges’ unilateral powers in mediation, a failed mediation is almost always followed by adjudication by the same judge, a feature that gives much more weight to judges’ suggestions and puts greater pressure on disputants (Huang, 2006; Ng & He, 2014). The highest success rate of mediations I observed was 95% in District Court C, the lowest being 85% in District Court B. These figures are similar to those found by scholars in other parts of China. In one study of victim–offender mediation in ten district courts in C city, Lu et al. (2017) found that the average success rate was around 91%. I found that almost all traffic accident cases can be settled within a half-day meeting, while fraud case is the toughest type for judges to handle.

Through my fieldwork in four district courts, I also uncovered that: (1) the SPC’s propaganda on national role models for female judges serves as an external force which has an impact on female judges’ preference for mediation over trials; (2) female judges’ recognition of the importance of resolving conflicts, gained from life and work experience, serves as an internal force which leads them to choose mediation as a preferred method of dispute resolution; (3) male judges neglect mediation; and (4) rape lawsuits are an exception, and are not mediated by female judges.

### The Impact of Propaganda

Chen Yanping, a female district court judge and national role model endorsed by the SPC, is a faithful follower of the Confucian ideal of “a world without litigation” (*tianxia wusong*). Official reports from the *People’s Court Daily* document that Judge Chen has handled over 3,100 cases in 14 years “without a single complaint or appeal; without a single petition by a disgruntled party; without even one wrongly decided case; her decisions are uniformly accepted by all parties” (He, 2010, as cited in Minzner, 2011, p. 950). In an interview, this national role model explained that her success stemmed from an avoidance of trials and her unflagging effort to mediate cases that came before her. Chen asserted that “judges should not be legal craftsmen who pay excessive attention to wording, believe the laws of statutes to be the only scripture, and pay no attention to social harmony and the popular interest” (Wang, 2010, p. 2, as cited in Minzner, 2011, p. 951). Although Chen worked in the civil division and her cases were predominantly civil disputes, her working style and spirit of benevolence also influenced district court judges in criminal divisions. According to my interviewees, Chen’s working style suited the SPC policy on “balancing leniency and severity” (*kuanyan xiangji*) in sentencing for serious crimes, whereas crimes with minimal social impact or mitigating circumstances are handled with relative leniency (Trevaskes, 2010, p. 332).

Under many authoritarian regimes, the media have played a critical role in the process of power consolidation (Stockmann & Gallagher, 2011). Although the propaganda work of the CPC in recent decades has been strengthened by introducing of a host of innovative new approaches, such as using the internet as a tool to fashion the CPC’s image, promoting politically constructed role models and moral exemplars remains a central tradition of propaganda. In fact, it would be surprising if the Chinese leadership did not continue to employ role models as a tool for political socialization and moral education. This is because, as Reed (1995) has pointed out, culture heroes are “one means through which the continuity of Chinese culture has been expressed over the centuries” (p. 99). For instance, in imperial China, chaste women were the subjects of commemorative writing by Confucian scholars and were the recipients of recognitions from central or local governments (Lu, 2010). Hundreds of chaste widows and faithful maidens were hence honored through the state-financed buildings of stone arches and memorial torii. Judge Chen is similar to Lei Feng, a soldier in the People’s Liberation Army and a communist legend in many aspects: loyalty, benevolence, and modesty, which are essential virtues of Confucianism. Admittedly, even though the image of Judge Chen and her predecessors could eventually fade from popular memory, it seems reasonable to speculate that the highest virtue of the Confucian role models will remain central to the process of socializing the Chinese people.

There are two key aspects of educational work in courts: one relates to propaganda work addressed in coordination with the mass media and the other relates to the court’s own propaganda work, which is undertaken independent of other state organs (Trevaskes, 2004). The *People’s Court Daily* is clearly the court’s own propaganda mouthpiece, and its stories are satisfying and attractive to judges who read them every day (Wei & Xin, 2013). It should be noted that the implicit message of these reports is that every judge is a potential model. If one imitates the publicized behaviors in accordance with the norms set by the SPC, one will be on the road to success. In my interviews, all the female judges claimed to have been influenced by Judge Chen and the exemplary work in mediation done by other female role models. They shared with me that when their divisions organized reading and sharing sessions on role models’ work, they were inspired by their devotion to the work. They held them up as women who have faced up to the challenges of the labor market and enjoyed success. In this way, they understood that under some circumstances court rulings may not fully settle disputes, and victims may not be properly compensated by offenders. Hence, they should pursue a win-win solution for all parties, accomplished through mediation.

Mere exposure to the media alone may not change people’s attitudes. Instead, the extent to which a person is influenced by the mass media depends on their level of awareness of a particular issue (Zaller, 1992). In the following section, I argue that female judges’ awareness and preference for victim–offender mediation have also been shaped by their life and work experience. Although I do not imply that all women have the same understanding of the law and the world, women do share a common cultural position in a society that is devalued relative to men and the masculine (Chafetz, 1990; Lorber, 1994). This experience might make them more sensitive to the plaintiff’s position and influence their behaviors (Martin, 1989).

### Reflection of Life and Work Experience

During my field work, some female judges reported that their life experience had taught them the benefits of fully settling disputes both at home and work. One junior judge (B-F-6) stated:

I had some fundamental disagreements with my husband on how to educate our children and allocate domestic responsibilities. It is true that family life can continue without addressing these significant differences. However, these disagreements were “ticking-time bombs” in my relationship. To restore the well-being of my family, I learned to deactivate these “bombs” as soon as I realized they existed. It is the same when I realize that my ruling may create a bigger conflict between the two parties of a lawsuit, I will do my best to settle such conflict even before the trial starts.

Another judge (D-F-2) shared her understanding of the function of mediation from her experiences of settling disputes with colleagues:

As a deputy division chief, I am not only expected to assist the chief in managing the division, I also need to handle workplace conflict, conflicts among my colleagues. I understand that such conflict rarely resolves itself. In fact, it normally escalates if not dealt with proactively and properly. In order to repair a broken relationship, I always serve a cup of tea to my colleagues before they sit down as a good gesture of attentiveness and building trust. I do exactly to same thing in mediation between litigants.

A common theme in these conversations is that judges believed life and work experience could help their judicial work and saw mediation as the best approach to settling disputes in the interests of litigants. Their behaviors indicate that they are more like problem-solvers than impartial arbitrators, and they focus on whether the broken relationship in a criminal lawsuit can be fixed. A study based on a unique dataset of 860 case records from a German trial court seems to support the finding that female judges might be more able to moderate and also be more empathetic regarding existing malfunctioning relationships between the two parties than their male counterparts. Specifically, Berlemann and Christmann (2019) found that female judges seem to exhibit higher settlement rates in long-term contractual relationships, whereas male judges perform better when the parties concur “by chance”: Tenancy cases typically originate from friction in the long-term relationship between the litigants, and female judges more often arrange settlements between the parties in such cases, whereas the relationships in tort law cases are typically somewhat coincidental. These gender-related differences in settlement probability and interpretation of the results merit our attention.

### Male Judges’ Neglect of Mediation

Female judges’ actions are not always mirrored by their male colleagues. Notably, all of the male judges interviewed agreed that Judge Chen’s work was impressive, claiming to see that her judicial style effectively settles disputes; however, most could not imagine employing her style in their own work, and only a few practiced mediations, and infrequently. A number of male judges spoke of abandoning mediation because of a belief that it is feminine. However, according to the *People’s Court Daily*, Judge Chen’s success was mostly a function of her “true heart, true sentiment and true love” (Wang, 2010, as cited in Minzner, 2011, p. 981). Male judges interpreted these qualities as “feministic conducts” which were not suitable for their work. Hence, they viewed female judges who presided over mediation as a matter of routine as women who actively performed their gender (Butler, 1990). This is perhaps why male judges sensed that conducting mediations would undermine their masculinity, “grouping” them with feminine work. One male judge (A-M-3) specifically mentioned the clothes which female judges wore in mediations:

I noticed that my female colleagues often wore casual outfits in mediations. I understand that these clothes made them more relatable, as sisters or aunties, and may make litigants feel comfortable in this process. However, judges are professionals, and that is why we have our robes. As a way to show my respect for this profession and the dignity of judicial office, I always wear my robe before the public. So I do not think the robe fits the setting of mediation.

When “casual outfits” were brought up by this male judge in interview, he undermined his female colleagues’ professional role as judges in the workplace and stressed their roles in everyday life. In linguistics, this is called indexicality, and it is a key component of the performance and manufacturing of identity (Hanks, 1999). Sunderland (2004) provided an example of a woman reminding her colleagues to wear a coat on a rainy day. Her colleagues respond, “Thanks, mom!” Without a common social understanding of the role of mothers, this comment would not make sense. However, against the backdrop of mothers as homemakers and caretakers, this response teases a female colleague for her adherence to traditional gender roles. Similarly, female judges’ referencing of “sister or aunties” connects those terms to a broader social reference point and helps people to index these phrases through humor or insult. Six male judges repeatedly mentioned that they were surprised to find that after Judge Chen was described as a national role model by the SPC, that each high court selected its own mediation role models and they “all happened to be women.” Since almost all the male judges interviewed, consciously or unconsciously, labeled victim–offender mediation as feminine, they argued that a performance of mediation would inevitably result in them being ridiculed by male colleagues. This is why they opt out of such an approach.

Another reason for male judges’ depreciation of mediation is that it is time-consuming and leaves judges less time to finish other cases on their dockets. According to my observations, a simple case takes on average 2.5 hours to complete, excluding time spent on preparation for the mediation and on producing the written agreement after mediation. This observation is similar to the findings of research conducted by Song et al. (2009) in eight district courts, where mediations lasted “an average of two hours, but this calculation excludes time spent with individual parties and prosecutors, and time spent writing outcome confirmation letters” (p. 9). However, for complex cases with multiple offenders and victims involved, judges need to carry out several rounds of mediations for reaching consensus between the parties, and each session may take up to three hours to complete. Since mediations require a large investment of time, five male judges similarly stated that they could not “find any reason to play a role in mediation because their work already stressful.”

### Rape Lawsuits as an Exception

Among the several types of crime which can be mediated according to 2018 Criminal Procedure Law, rape is an exception in practice. Female judges in four district courts uniformly refuse to mediate rape lawsuits, and two gave distinct reasons for their choice:

I have worked in this division for 15 years, and it is extremely difficult for me to invite rape victims to speak with their offender face to face. I know they have gone through this before with police in investigation and prosecutors in prosecution, and I can see the suffering they experienced in telling their stories again and again. Because their shame is so deep, some even choose not to come forward to us. That is why I hope rape cases can always be closed efficiently through trials, instead of going through the lengthy process of mediation.

I am a woman and was sexually assaulted when I was at boarding school. I still remember that nights were difficult for me, and I had to double-check the door and windows of my room before I went to bed. When I heard footsteps outside my room, I had to turn on the light to show I was not asleep. You see, I understand how hard it is for a girl who has experienced this, and it must be a tremendous setback to her life. That is why I have never mediated a single rape case in my career—one case is too many.

It is well documented that members of the criminal justice system share society’s bias against rape victims (Du Mont et al., 2003; Maier, 2008). As a result, a victim may have to endure repetitive questioning about the rape itself as well as about her relationship with the rapist. This insensitive treatment by police and prosecutors may magnify feelings of powerlessness and shame in victims, produce feelings of guilt and lower self-esteem (Patterson & Campbell, 2010). The reason members of the criminal justice system frequently overlook the rights and needs of the victims is that they view complainants as “just another piece of evidence”—the victim’s role is to establish a legal case against the offender (Bohmer, 1973, p. 303). This is why victims regularly lack control of their situations and report that their encounters with police and prosecutors were more traumatic than the rape incident itself.

It seems that these female judges take the suffering of rape victims and their own experiences into consideration in refusing to mediate rape lawsuits. This is possible because women are most frequently the victims of sexual assault, and their personal experience of assault may make it easier for them to identify with victims of assault than for men (Pryor & Day, 1988; Rotundo et al., 2001). This is also in line with what Li (2007) found through surveying and interviewing 136 judges in two regions of China: They all recognized that judges’ own experiences and external factors had an impact on their decision-making process in criminal lawsuits. The judges’ own experiences in Li’s research were of assault and maltreatment in their own lives, which is particularly significant in sex crimes. Li also reported that female judges took the harm inflicted upon victims as the most important factor in sentencing, while male judges did not consider victims’ experiences as a critical factor. Rather, they preferred to take a holistic view of sex crimes when determining the length of a sentence. Regrettably, since there is a very limited number of male judges conducting mediation in four district courts, their thoughts on mediating rape lawsuits could not be addressed here.

## Discussion

Since the SPC began promoting mediation in courts in 2005, judges have questioned to what degree it can be used to resolve disputes. I observed judges in the four district courts to be divided along gender lines on this issue, with more female judges mediating cases and male judges refusing to use mediation. Female judges pursued mediation in large part due to the influence of Judge Chen, the national role model. At the same time, in interviews they expressed the view that their life and work experience taught them the benefits of fully settling disputes. Conversely, male judges believed that using mediation would significantly undermine their masculinity. They also had concerns about the efficiency of mediation, believing that similar outcomes could be reached by trial. The male judges’ attitudes toward mediation can be explained by sex role spillover theory, which relates to the carryover of gender-based expectations of behavior in the workplace (Gutek & Morasch, 1982). According to this theory, numerically dominant men are likely to see non-traditionally employed women as women first and bearers of a work role second (Gutek & Cohen, 1987). Therefore, women’s contributions and work behaviors may be viewed and valued differently from those of their male colleagues. Sex role spillover theory has been used widely to examine the harmful effects of discrimination and the increasing participation rates of women in the workforce. For instance, Luksyte et al. (2018) found that innovative work behavior is stereotypically ascribed more to men than to women. Building on this finding, their study shows that when men and women similarly engaged in innovative work, men experienced greater returns than women in terms of performance appraisal. To apply this theory to this study, it can be observed that all the male judges noted that it was Judge Chen and her followers, who were predominantly female, who actively used mediation as their preferred dispute settlement method. Because of this, male judges connected mediation to feminine characteristics rather than work role requirements. Since “the traits associated with the stereotype of women are not particularly valued in some workplaces,” almost all male judges declined to adopt mediation as a method of dispute resolution (Gutek & Morasch, 1982, p. 99).

My fieldwork indicates that because of female judges’ active facilitation of victim–offender mediation, most victims received apologies and compensation from offenders as a result of female judges’ time and effort. This contribution has unique significance if examined through a historical lens. Peerenboom (1993) surveyed the role of the victim in premodern Chinese legal practice and found that the formal legal system is concerned primarily with the interests of the state and society as a whole. Accordingly, the system is much more concerned with punishing the offender than catering to the psychological and emotional needs of the victim. Even the socialist criminal system has its primary task as the prevention and punishment of crimes that seriously disrupt the public order, instead of focusing on the needs and concerns of the victim. In the last decade, China has witnessed growth in the protection of the offender’s rights and a recurring lack of attention to the victim. Research suggests that when an incidental civil action is heard together with a criminal case, the procedures relating to the criminal aspects of the case predominate, and the procedures designed to adjudicate upon the civil component of the case are simplified or even ignored (McConville, 2011). However, the design of victim–offender mediation in 2012 offered for the first time an opportunity for the victim to play an active role in shaping the outcome of a lawsuit. To a certain extent, it alleviates victims’ suffering by empowering them to influence or even determine the offender’s destiny. My fieldwork revealed that it is female judges who constantly manage to promote the offender’s accountability and responsibility, repair the harm caused by their criminal behaviors, and meet the interests of victims. This “gendered justice” will be a new arena for the academic discussion of restorative justice in China.

## Conclusion

In her study of American women in the legal profession from a cross-cultural perspective, Menkel-Meadow (1989) scrutinized techniques and strategies of exclusion and developed the notion of the “glass ceiling for practicing women” (p. 295). Agreeing with Gilligan’s (1982) view, Menkel-Meadow and other feminist legal scholars continued to explore the essential differences between the two genders. However, the conflicting results demonstrate the difficulties of locating an essential women’s difference in judging (Kenney, 2012). Instead of focusing on statistical differences between men and women, a few scholars have taken a methodological turn and examined the judicial behaviors of male and female judges in practice. They have found that female judges consider mediation a preferred method in settling disputes (Boyd, 2013; Ludewig & LaLlave, 2013). Following this line of thought, the present research examined victim–offender mediation, which is permissive but not mandatory in 2018 Criminal Procedure Law, in four Chinese district courts. I found that, similar to the findings in the literature, female judges in these district courts used victim–offender mediation as a means to seek apology from offenders and compensation for victims. The choice of mediation is the result of a combination of propaganda from the SPC and a reflection of female judges’ life and work experience. For research on gender, law, and judging, this study is groundbreaking in two aspects: First, it reveals that in the Chinese criminal justice system, behavioral differences between male and female judges exist in the process rather than the outcome of judgment. The literature focuses solely on male and female Chinese judges’ differences in conviction and sentencing, without probing other working styles that can also settle disputes in criminal cases (Wei & Xiong, 2020). Second, this research reveals male and female judges’ different understandings of the function of mediation. These differences can help researchers understand why male and female judges choose different methods to settle legal disputes. Undoubtedly, it is within the context of a growing body of research on gender, law, and judging that this study gains its significance because this Chinese case study can speak to the feminist legal studies in Western society yet retains its own complexity and specificity. However, like many ethnographic projects examining the Chinese criminal justice system, this research is constrained by the duration of the fieldwork and the locations of the selected courts. Therefore, these contributions should be understood within the context of the following important limitations.

First, this study reveals that female judges are influenced by the SPC’s propaganda and tend to use victim–offender mediation as a preferred dispute settlement method, as their role models do. I cautiously remind the reader that such judicial behaviors are influenced by and are a result of “grand mediation” (*da tiaojie*). The propaganda of the CPC regarding judicial policies could shift to other focuses in years to come, and the influence of role model female judges could diminish. Hence, it is possible that the preference for victim–offender mediation might be discarded by female judges in the future. Second, this research focuses on the intersection of gender and judicial behaviors, so it is vital to recognize the impact of my own male gender on interviewees’ responses. In the literature, after interviewing trial court judges in Indiana, Artis (2004) reflected that because of her identity as a female researcher, male judges “may not have felt comfortable discussing the gendered components of custody disputes” (p. 781) with her. I felt the same constraint when engaging in conservations with male judges on their neglect of victim–offender mediation. While Schwalbe and Wolkomir (2001) highlighted that the reciprocal enactment of masculinity within an all-male interview context can actually facilitate dialogue and a depth that may not otherwise be possible, I sensed that male judges considered their words very carefully before me in order not to be viewed as biased against their female colleagues. I understand that since the individual biography of the qualitative researcher is recognized to have a major impact on research projects and respondents, every insight into the social world drawn from interviews is inevitably partial (Gelsthorpe, 1992). It is therefore expected that a replication of this project by a female researcher would enable those who analyze the results afterwards to compare the narrative accounts offered by participants as well as the play of gender dynamics in interviews.

China is a country of such immense size and contrasts that one project cannot provide a fully representative sample of cases. As a result, a natural extension of this research would be to visit district courts in remote areas in order to bring more geographic differences into focus. Scholars have found that mediation is frequently used by villagers to settle disputes in rural China, especially in ethnic minority areas (Zhu, 2016). This is largely because rural disputants are more likely to be acquaintances. They thus tend to avoid formal laws to preserve the existing interpersonal web that villagers rely on (Di & Wu, 2009). In rural areas, male and female judges in criminal divisions could be similarly inspired by local cultures and practices to employ mediation in their work. Since the workload of judges in such areas may be lighter, they might devote more time to fostering agreement between parties through mediation. Further research could also include interviews with victims and offenders on their feelings about mediation. It has been indicated in this study that the success rates of victim–offender mediation in the four district courts is high. However, both parties’ innate reactions to the quality of mediation remains unknown because their overt compliance in mediation could simply be due to a wish to avoid extreme decisions in a trial presided by the same judge. In this criminological fieldwork, I was constrained by my research ethics to interview victims and offenders after the mediation because both were unable to give free consent until their case had been fully settled. Although I frequently saw them shake hands with the judges, I did not know whether they were truly satisfied with the outcome of their mediation.

Finally, I invite feminist legal scholars to reassess the research methods of exploring the idea of a “different voice” in decision making. One of the major criticisms of qualitative research is its lack of scientific rigor (Mays & Pope, 1995). Nevertheless, it is through qualitative research that female Chinese judges’ preference for victim–offender mediation was uncovered by this project. Moreover, their narratives and biographies offer rich insights and provide accurate information for analysis by follow-up studies. As the field of gender, law, and judging moves forward, the qualitative approach may be able to address more difficult questions, such as a better approach for supporting victims in the criminal justice system. To conclude, although women in our time no longer need to dress like men to argue before the court, as Portia did in Shakespeare’s play, the gap between men and women in the legal profession remains, and the barriers for women to overcome have become subtler (Schultz & Shaw, 2013; Thornton, 1996). It is generally agreed that the strength of the legal profession lies in its equality and diversity. Having more women as equal partners of male judges for a diverse and representative judiciary not only enriches its openness to viewpoints previously marginalized but underscores its legitimacy as one of the most powerful institutions (Kenney, 2012; Rackley, 2012). I therefore look forward to more qualitative studies that can bring out women’s experiences in this area and empower women through their unique “different voice.”

## Appendix

**Table:** Demographics of Judges (*N* = 42).

Characteristics	Number	Percentage
Gender		
Male	27	64
Female	15	36
Age		
23–30	7	17
31–40	19	45
41–50	12	28
51–60	4	10
Education		
Bachelor’s	19	45
Master’s	17	40
Doctoral	6	15
Bar admission		
Yes	31	74
No	11	26
Years on bench		
10 years or less	10	24
11–20 years	21	50
21–30 years	9	21
31 years or above	2	5

## Notes

1. A common distinction between sex and gender differentiates sex as biological characteristics and gender as a social construction, although such binary classifications have been criticized as counterproductive. In discussion of women’s representation in the judiciary and their behaviors, it is the term “gender” that is frequently adopted by legal scholars. I follow this practice.

2. The difference between victim–offender mediation and criminal reconciliation is that the former emphasizes the participation of a third, neutral party who presides over such meetings while the latter refers to a situation where offender and victim meet in a non-adversarial environment to search for solutions that promote reparation.

3. A refers to District Court A in Shanghai; M and F are used to identify judges’ gender; and Arabic numerals differentiate judges interviewed in the same court.

## References

[bibr1-0306624X20936202] Armstrong G. (1977). Females under the law—“Protected” but unequal. Crime & Delinquency, 23(2), 109–120.

[bibr2-0306624X20936202] Artis J. E. (2004). Judging the best interests of the child: Judges’ accounts of the tender year doctrine. Law & Society Review, 38(4), 769–806.

[bibr3-0306624X20936202] Auerbach J. Blum L. Smith V. Williams C. (1985). On Gilligan’s “In a different voice.” Feminist Studies, 11(1), 149–161.

[bibr4-0306624X20936202] Baldwin J. (2000). Research on the criminal courts. In King R. Wincup E. (Eds.), Doing research on crime and justice (pp. 375–398). Oxford University Press.

[bibr5-0306624X20936202] Bennett R. R. (2004). Comparative criminology and criminal justice research: The state of our knowledge. Justice Quarterly, 21(1), 1–21.

[bibr6-0306624X20936202] Berlemann M. Christmann R. (2019). Determinants of in-court settlements: Empirical evidence from a German trial court. Journal of Institutional Economics, 15(1), 143–162.

[bibr7-0306624X20936202] Bodde D. Morris C. (1967). Law in imperial China: Exemplified by 190 Ch’ing dynasty cases: With historical, social, and juridical commentaries. Harvard University Press.

[bibr8-0306624X20936202] Bohmer C. (1973). Judicial attitudes toward rape victims. Judicature, 57, 303–307.

[bibr9-0306624X20936202] Bottoms A. (2000). The relationship between theory and research in criminology. In King R. Wincup E. (Eds.), Doing research on crime and justice (pp. 15–60). Oxford University Press.

[bibr10-0306624X20936202] Boyd C. L. (2013). She’ll settle it? Journal of Law and Courts, 1(2), 193–219.

[bibr11-0306624X20936202] Boyd C. L. Epstein L. Martin A. D. (2010). Untangling the causal effects of sex on judging. American Journal of Political Science, 54(2), 389–411.

[bibr12-0306624X20936202] Bratton K. A. (2005). Critical mass theory revisited: The behavior and success of token women in state legislatures. Politics & Gender, 1(1), 97–125.

[bibr13-0306624X20936202] Brockman J. (2001). Gender in the legal profession: Fitting or breaking the mould. UBC Press.

[bibr14-0306624X20936202] Butler J. (1990). Gender trouble. Routledge.

[bibr15-0306624X20936202] Chafetz J. S. (1990). Gender equity: An integrated theory of stability and change (Vol. 176). Sage Publications.

[bibr16-0306624X20936202] Cohen J. A. (1966). Chinese mediation on the eve of modernization. California Law Review, 54(3), 1201–1226.

[bibr17-0306624X20936202] Coontz P. (2000). Gender and judicial decisions: Do female judges decide cases differently than male judges? Gender Issues, 18(4), 59–73.

[bibr18-0306624X20936202] Curran D. J. (2010). Criminological research in China. In Cao L. Q. Sun I. Y. Hebenton B. (Eds.), The Routledge handbook of Chinese criminology (pp. 160–170). Routledge.

[bibr19-0306624X20936202] Davis D. S. Harrell S. (Eds.). (1993). Chinese families in the post-Mao era (Vol. 17). University of California Press.

[bibr20-0306624X20936202] Davis S. Haire S. Songer D. R. (1993). Voting behavior and gender on the US courts of appeals. Judicature, 77, 129–133.

[bibr21-0306624X20936202] Di X. H. Wu Y. N. (2009). The developing trend of the people’s mediation in China. Sociological Focus, 42(3), 228–245.

[bibr22-0306624X20936202] Dinovitzer R. Hagan J. (2014). Hierarchical structure and gender dissimilarity in American legal labor markets. Social Forces, 92(3), 929–955.

[bibr23-0306624X20936202] Du Mont J. Miller K. L. Myhr T. L (2003). The role of “real rape” and “real victim” stereotypes in the police reporting practices of sexually assaulted women. Violence Against Women, 9(4), 466–486.

[bibr24-0306624X20936202] Dutton M. R. (1992). Policing and punishment in China: From patriarchy to “the people” (Vol. 141). Cambridge University Press.

[bibr25-0306624X20936202] Fineman M. A. Thomadsen N. S. (Eds.). (2013). At the boundaries of law (RLE feminist theory): Feminism and legal theory. Routledge.

[bibr26-0306624X20936202] Galanter M. (1985). The emergence of the judge as a mediator in civil cases. Judicature, 69(5), 257–262.

[bibr27-0306624X20936202] Gelsthorpe L. (1992). Response to Martyn Hammersley’s paper on feminist methodology. Sociology, 26(2), 213–218.

[bibr28-0306624X20936202] Gilligan C. (1982). In a different voice. Harvard University Press.

[bibr29-0306624X20936202] Goh B. C. (2016). Law without lawyers, justice without courts: On traditional Chinese mediation. Routledge.

[bibr30-0306624X20936202] Goolsby D. G. (1990). Using mediation in cases of simple rape. Washington & Lee Law Review, 47(4), 1183–1214.

[bibr31-0306624X20936202] Gottschall J. (1983). Carter’s judicial appointments: The influence of affirmative action and merit selection on voting on the US courts of appeals. Judicature, 67(4), 165–174.

[bibr32-0306624X20936202] Gray D. E. (2013). Doing research in the real world. Sage.

[bibr33-0306624X20936202] Gruhl J. Spohn C. Welch S. (1981). Women as policymakers: The case of trial judges. American Journal of Political Science, 25(2), 308–322.

[bibr34-0306624X20936202] Gutek B. A. Cohen A. G. (1987). Sex ratios, sex role spillover, and sex at work: A comparison of men’s and women’s experiences. Human Relations, 40(2), 97–115.

[bibr35-0306624X20936202] Gutek B. A. Morasch B. (1982). Sex-ratios, sex-role spillover, and sexual harassment of women at work. Journal of Social Issues, 38(4), 55–74.

[bibr36-0306624X20936202] Hanks W. (1999). Indexicality. Journal of Linguistic Anthropology, 9(1/2), 124–126.

[bibr37-0306624X20936202] He C. Z. (2010). A rural basic level people’s judge whose decisions are accepted by victors and losers alike. http://zqb.cyol.com/content/2010-01/18/content_3043751.htm

[bibr38-0306624X20936202] Heimer M. Thøgersen S. (Eds.). (2006). Doing fieldwork in China. University of Hawaii Press.

[bibr39-0306624X20936202] Huang P. C. (2006). Court mediation in China, past and present. Modern China, 32(3), 275–314.

[bibr40-0306624X20936202] Ji Y. (2015). Between tradition and modernity: “Leftover” women in Shanghai. Journal of Marriage and Family, 77(5), 1057–1073.

[bibr41-0306624X20936202] Jiang S. (2017). From “harsh justice” to “balancing leniency with severity.” Peking University Law Journal, 5(1), 139–164.

[bibr42-0306624X20936202] Jiang S. H. Wu Y. N. (2015). Chinese people’s intended and actual use of the court to resolve grievance/dispute. Social Science Research, 49, 42–52. 25432602 10.1016/j.ssresearch.2014.07.009

[bibr43-0306624X20936202] Johnson B. D. (2006). The multilevel context of criminal sentencing: Integrating judge- and county-level influences. Criminology, 44(2), 259–298.

[bibr44-0306624X20936202] Johnson E. H. (1986). Politics, power and prevention: The People’s Republic of China case. Journal of Criminal Justice, 14(5), 449–457.

[bibr45-0306624X20936202] Kenney S. J. (2008). Thinking about gender and judging. International Journal of the Legal Profession, 15(1–2), 87–110.

[bibr46-0306624X20936202] Kenney S. J. (2012). Gender and justice: Why women in the judiciary really matter. Routledge.

[bibr47-0306624X20936202] Klein K. K. (2005). A judicial mediator’s perspective: The impact of gender on dispute resolution: Mediation as a different voice. North Dakota Law Review, 81(4), 771–794.

[bibr48-0306624X20936202] Kritzer H. M. Uhlman T. M. (1977). Sisterhood in the courtroom: Sex of judge and defendant in criminal case disposition. Social Science Journal, 14(2), 77–88.

[bibr49-0306624X20936202] Kronman A. T. (1995). The lost lawyer: Failing ideals of the legal profession. Harvard University Press.

[bibr50-0306624X20936202] Leung A. S. M. (2003). Feminism in transition: Chinese culture, ideology and the development of the women’s movement in China. Asia Pacific Journal of Management, 20(3), 359–374.

[bibr51-0306624X20936202] Lewis J. W. Litai X. (2003). Social change and political reform in China: Meeting the challenge of success. The China Quarterly, 176, 926–942.

[bibr52-0306624X20936202] Li L. (2017). The Chinese Communist Party and people’s courts: Judicial dependence in China. In Brodsgaard K. E. (Ed.), Critical readings on Communist Party of China (pp. 1320–1368). Brill.

[bibr53-0306624X20936202] Li R. (2007). Yingxiang xingshi faguan panjue yinsu de shizheng Kkaocha [On positive investigation about elements influencing criminal judges’ trial]. Law Science Magazine, 6, 96–98.

[bibr54-0306624X20936202] Liebman B. L. (2011). A populist threat to China’s courts? In Woo M. Y. Gallagher M. E. (Eds.), Chinese justice: Civil dispute resolution in contemporary China (pp. 269–271). Cambridge University Press.

[bibr55-0306624X20936202] Littleton C. A. (1986). Equality and feminist legal theory. University of Pittsburgh Law Review, 48, 1043–1060.

[bibr56-0306624X20936202] Lorber J. (1994). Paradoxes of gender. Yale University Press.

[bibr57-0306624X20936202] Lu H. Li Y. Liang B. (2018). Restorative justice and probation decisions—An analysis of intentional assault cases in China. Psychology, Crime & Law, 24(6), 573–588.

[bibr58-0306624X20936202] Lu W. J. (2010). The chaste and the licentious: Female sexuality and moral discourse in Ming and early Qing China. Early Modern Women, 5, 183–187.

[bibr59-0306624X20936202] Lu Z. X. Ke J. Ou M. Y. (2017). Xingshi hejie shenpan chengxu zhi xianshi chujing yu wanshan jinlu—Jiyu xin xingshi susongfa shishihou yunxing zhuangkuang de shizheng kaocha [The current status of victim–offender mediation and its path forward: An empirical study based on the implementation of new criminal procedure law]. Journal of Law Application, 11, 76–83.

[bibr60-0306624X20936202] Ludewig R. LaLlave J. (2013). Professional stress, discrimination and coping strategies: Similarities and differences between female and male judges in Switzerland. In Schultz U. Shaw G. (Eds.), Gender and judging (pp. 233–252). Hart Publishing.

[bibr61-0306624X20936202] Luksyte A. Unsworth K. L. Avery D. R. (2018). Innovative work behavior and sex-based stereotypes: Examining sex differences in perceptions and evaluations of innovative work behavior. Journal of Organizational Behavior, 39(3), 292–305.

[bibr62-0306624X20936202] MacKinnon C. A. (1983). Feminism, Marxism, method, and the state: Toward feminist jurisprudence. Signs: Journal of Women in Culture and Society, 8(4), 635–658.

[bibr63-0306624X20936202] Maier S. L. (2008). “I have heard horrible stories. . .”: Rape victim advocates’ perceptions of the revictimization of rape victims by the police and medical system. Violence Against Women, 14(7), 786–808. 18559867 10.1177/1077801208320245

[bibr64-0306624X20936202] Mann S. L. (2011). Gender and sexuality in modern Chinese history. Cambridge University Press.

[bibr65-0306624X20936202] Martin E. (1989). Men and women on the bench: Vive la difference. Judicature, 73(4), 204–208.

[bibr66-0306624X20936202] Martin E. Pyle B. (1999). Gender, race, and partisanship on the Michigan Supreme Court. Albany Law Review, 63(4), 1205–1236.

[bibr67-0306624X20936202] Martin E. Pyle B. (2004). State high courts and divorce: The impact of judicial gender. University of Toledo Law Review, 36(4), 923–948.

[bibr68-0306624X20936202] Mays N. Pope C. (1995). Qualitative research: Rigour and qualitative research. BMJ, 311(6997), 109–112. 7613363 10.1136/bmj.311.6997.109 PMC2550154

[bibr69-0306624X20936202] McCall M. (2003). Gender, judicial dissent, and issue salience: The voting behavior of State Supreme Court justices in sexual harassment cases, 1980–1998. The Social Science Journal, 40(1), 79–97.

[bibr70-0306624X20936202] McConville M. (2011). Criminal justice in China: An empirical inquiry. Edward Elgar Publishing.

[bibr71-0306624X20936202] Menkel-Meadow C. (1989). Exploring a research agenda of the feminization of the legal profession: Theories of gender and social change. Law & Social Inquiry, 14(2), 289–319.

[bibr72-0306624X20936202] Miller S. L. Maier S. L. (2008). Moving beyond numbers: What female judges say about different judicial voices. Journal of Women, Politics & Policy, 29(4), 527–559.

[bibr73-0306624X20936202] Min D. H. (2011). From men-women equality to gender equality: The zigzag road of women’s political participation in China. Asian Journal of Women’s Studies, 17(3): 7–24.

[bibr74-0306624X20936202] Minow M. (1987). Foreword: Justice engendered. Harvard Law Review, 101, 10–95.

[bibr75-0306624X20936202] Minzner C. F. (2011). China’s turn against law. The American Journal of Comparative Law, 59(4), 935–984.

[bibr76-0306624X20936202] Nader L. (1993). Controlling processes in the practice of law: Hierarchy and pacification in the movement to reform dispute ideology. Ohio State Journal on Dispute Resolution, 9(1), 1–26.

[bibr77-0306624X20936202] National Bureau of Statistics of People’s Republic of China. (2015). Statistical report on the implementation of the Chinese women development outline (2011–2020) in 2013. http://www.stats.gov.cn/tjsj/zxfb/201501/t20150122_672472.html

[bibr78-0306624X20936202] Ng K. H. He X. (2014). Internal contradictions of judicial mediation in China. Law & Social Inquiry, 39(2), 285–312.

[bibr79-0306624X20936202] Parish W. L. Farrer J. (2000). Gender and family. In Tang W. Parish W. L. (Eds.), Chinese urban life under reform (pp. 232–272). Cambridge University Press.

[bibr80-0306624X20936202] Patterson D. Campbell R. (2010). Why rape survivors participate in the criminal justice system. Journal of Community Psychology, 38(2), 191–205.

[bibr81-0306624X20936202] Peerenboom R. P. (1993). The victim in Chinese criminal theory and practice: A historical survey. Journal of Chinese Law, 7(1), 63–110.

[bibr82-0306624X20936202] Pryor J. B. Day J. D. (1988). Interpretations of sexual harassment: An attributional analysis. Sex Roles, 18(7–8), 405–417.

[bibr83-0306624X20936202] Rackley E. (2012). Women, judging and the judiciary: From difference to diversity. Routledge-Cavendish.

[bibr84-0306624X20936202] Ramji-Nogales J. Schoenholtz A. I. Schrag P. G. (2007). Refugee roulette: Disparities in asylum adjudication. Stanford Law Review, 60(2), 295–412.

[bibr85-0306624X20936202] Reed G. G. (1995). Moral/political education in the People’s Republic of China: Learning through role models. Journal of Moral Education, 24(2), 99–111.

[bibr86-0306624X20936202] Rotundo M. Nguyen D. H. Sackett P. R. (2001). A meta-analytic review of gender differences in perceptions of sexual harassment. Journal of Applied Psychology, 86(5), 914–922. 10.1037/0021-9010.86.5.914 11596807

[bibr87-0306624X20936202] Schultz U. Shaw G. (Eds.). (2013). Gender and judging. Bloomsbury Publishing.

[bibr88-0306624X20936202] Schwalbe M. Wolkomir M. (2001). The masculine self as problem and resource in interview studies of men. Men and Masculinities, 4(1), 90–103.

[bibr89-0306624X20936202] Shen A. Q. (2017). Women judges in contemporary China: Gender, judging and living. Springer.

[bibr90-0306624X20936202] Silbey S. S. Merry S. E. (1986). Mediator settlement strategies. Law & Policy, 8(1), 7–32.

[bibr91-0306624X20936202] Smith F. Jr. (2004). Gendered justice: Do male and female judges rule differently on questions of gay rights. Stanford Law Review, 57(6), 2087–2134.

[bibr92-0306624X20936202] Song L. S. Liu F. Q. (2016). Falv zhiye zhong de nvxing: Cong faxueyuan dao fayuan [Women in the legal profession: From law school to court]. Law and Social Sciences, 15(2), 166–192.

[bibr93-0306624X20936202] Song L. S. Xie T. (2014). “Haofaguan” de juese dingwei jiqi zhishi puxi—Yi nvxing mingshi faguan wei yangben de shizheng yanjiu [The position and understanding of a “good judge”—An empirical study based on female judges in civil divisions.]. Journal of Shanghai University of Political Science & Law, 29(3), 10–16.

[bibr94-0306624X20936202] Song Y. H. Guo Y. Z. Li Z. Luo H. M. He T Lei X. Z. Xiang Y. Wang Z. H. Feng S. F. (2009). Gongsu anjian xingshi hejie shizheng yanjiu [An empirical study to victim–offender mediation of prosecuted lawsuits]. Chinese Journal of Law, 3, 3–22.

[bibr95-0306624X20936202] Songer D. R. Crews-Meyer K. A. (2000). Does judge gender matter? Decision making in State Supreme Courts. Social Science Quarterly, 81(3), 750–762.

[bibr96-0306624X20936202] Songer D. R. Davis S. Haire S. (1994). A reappraisal of diversification in the federal courts: Gender effects in the Courts of Appeals. The Journal of Politics, 56(2), 425–439.

[bibr97-0306624X20936202] Sonneveld N. Lindbekk M. (2017). Women judges in the Muslim world: A comparative study of discourse and practice. Brill.

[bibr98-0306624X20936202] Spohn C. (1991). Decision making in sexual assault cases: Do black and female judges make a difference? Women & Criminal Justice, 2(1), 83–105.

[bibr99-0306624X20936202] State Council Information Office. (2019). Equal, development and sharing: The progress of women’s career in the 70 years since the establishment of the People’s Republic of China. http://www.xinhuanet.com/politics/2019-09/19/c_1125015082.htm

[bibr100-0306624X20936202] Stockmann D. Gallagher M. E. (2011). Remote control: How the media sustain authoritarian rule in China. Comparative Political Studies, 44(4), 436–467.

[bibr101-0306624X20936202] Su Y. He X. (2010). Street as courtroom: State accommodation of labor protest in South China. Law & Society Review, 44(1), 157–184.

[bibr102-0306624X20936202] Sunderland J. (2004). Gendered discourses. Palgrave Macmillan.

[bibr103-0306624X20936202] Thornton M. (1996). Dissonance and distrust: Women in the legal profession. Oxford University Press.

[bibr104-0306624X20936202] Tong X. N. Tang S. H. (2015). Yunling role model Yu Huafen: Deal with every case diligently and faithfully. http://rmfyb.chinacourt.org/paper/images/2015-08/25/05/2015082505_pdf

[bibr105-0306624X20936202] Trevaskes S. (2004). Propaganda work in Chinese courts: Public trials and sentencing rallies as sites of expressive punishment and public education in the People’s Republic of China. Punishment & Society, 6(1), 5–21.

[bibr106-0306624X20936202] Trevaskes S. (2010). The shifting sands of punishment in China in the era of “harmonious society.” Law & Policy, 32(3), 332–361.

[bibr107-0306624X20936202] Trevaskes S. Nesossi E. Sapio F. Biddulph S. (Eds.). (2014). The politics of law and stability in China. Edward Elgar Publishing.

[bibr108-0306624X20936202] Troyer R. J. (1989). Chinese thinking about crime and social control. In Troyer R. J. Clark J. P. Rojek D. G. (Eds.), Social control in the People’s Republic of China (pp. 45–56). Praeger http://www.ncjrs.gov/App/publications/abstract.aspx?ID=120034

[bibr109-0306624X20936202] Walker T. G. Barrow D. J. (1985). The diversification of the federal bench: Policy and process ramifications. The Journal of Politics, 47(2), 596–617.

[bibr110-0306624X20936202] Wang P. (2014). Extra-legal protection in China: How guanxi distorts China’s legal system and facilitates the rise of unlawful protectors. British Journal of Criminology, 54(5), 809–830.

[bibr111-0306624X20936202] Wang Z. C. (2010). An exemplary person should not become a vessel: The judicial wisdom of Chen Yanping. http://rmfyb.chinacourt.org/paper/html/2010-02/09/content_4032.htm

[bibr112-0306624X20936202] Weatherley R. Pittam H. (2015). Money for Life: The legal debate in China about criminal reconciliation in death penalty cases. Asian Perspective, 39(2), 277–299.

[bibr113-0306624X20936202] Wei S. Xin X. (2013). Does gender play a role in divorce mediation?: Working pattern of women judges in China. Asian Journal of Women’s Studies, 19(3), 149–171.

[bibr114-0306624X20936202] Wei S. Xiong M. L. (2020). Judges’ gender and sentencing in China: An empirical inquiry. Feminist Criminology, 15(2), 217–250.

[bibr115-0306624X20936202] Wu Y. N. (2014). People’s mediation in China. In Cao L. Sun I. Hebenton B. (Eds.), The Routledge handbook of Chinese criminology (pp. 116–129). Routledge.

[bibr116-0306624X20936202] Xiang Y. (2013). Criminal mediation in mainland China: A leap from judicial endeavor to legal norm. Asian Journal of Criminology, 8(4), 247–256.

[bibr117-0306624X20936202] Xiong M. Wei S. (2017). Unequal treatment in pretrial detention in China. The British Journal of Criminology, 57(6), 1398–1419.

[bibr118-0306624X20936202] Yang J. L. (2016). The life story of zhouxin: A role model female judge from the criminal division of Shanghai Intermediate People’s Court. http://rmfyb.chinacourt.org/paper/images/2016-01/26/05/2016012605_pdf

[bibr119-0306624X20936202] Yao S. (1999). Economic growth, income inequality and poverty in China under economic reforms. The Journal of Development Studies, 35(6), 104–130.

[bibr120-0306624X20936202] Yuan X. Y. (2017). Restorative justice in China: Comparing theory and practice. Springer.

[bibr121-0306624X20936202] Yuan X. Y. (2018). Conducting criminological fieldwork in China: A *guanxi* approach? Asian Journal of Criminology, 13(2), 79–90.

[bibr122-0306624X20936202] Zaller J. R. (1992). The nature and origins of mass opinion. Cambridge University Press.

[bibr123-0306624X20936202] Zhang L. Messner S. F. Liu J. Zhuo Y. A. (2009). *Guanxi* and fear of crime in contemporary urban China. The British Journal of Criminology, 49(4), 472–490.

[bibr124-0306624X20936202] Zheng C. Y. Ai J. H. Liu S. D. (2017). The elastic ceiling: Gender and professional career in Chinese courts. Law & Society Review, 51(1), 168–199.

[bibr125-0306624X20936202] Zhu S. L. (2012). Zhongguo faguan de xingxiang suzao [The framing of Chinese judges]. Legal Information, 1, 4–7.

[bibr126-0306624X20936202] Zhu S. L. (2016). Sending law to the countryside. Springer.

[bibr127-0306624X20936202] Zhuo Y. Cao L. Q. (2016). Intended and actual use of civil dispute resolution in contemporary China. Crime, Law and Social Change, 66(5), 507–523.

[bibr128-0306624X20936202] Zuo W. M. (2018). Susong baozha de zhongguo yingdui: Jiyu W fayuan jin sanshinian shenpan shijian de shizheng fenxi [The Chinese approach of handling litigation explosions: An empirical study to W District Court for the past thirty years]. China Legal Science, 4, 238–260.

